# Perfect combination of the expanded flap and 3D printing technology in reconstructing a child’s craniofacial region

**DOI:** 10.1186/s13005-020-00219-1

**Published:** 2020-03-03

**Authors:** Yanni Wang, Hongyan Qi

**Affiliations:** Department of Burn and Plastic Surgery, Beijing Children’s Hospital, No. 56 Nanlishi Road, Xicheng District, Beijing, 100045 China

**Keywords:** Expanded flap, 3D printing, Craniofacial region

## Abstract

**Background:**

The reconstruction of large head and face missing structures in the craniofacial region in children is very challenging for plastic surgeons. Expanded local and expanded axial-pattern flaps are widely used for the reconstruction of large-area scars. Free flaps are used very cautiously in children. 3D printing technology is a new technology with great development potential. 3D printing technology is used to assist in individualizing titanium alloy restorations for prefabricated skull defect repair. This application has great advantages in the repair of large skull loss. However, it is crucial to choose appropriate techniques and treat deformities of the head and face with integrated approaches and collaboration among multiple departments.

**Case presentation:**

This study proposes a method to combine the expanded flap method and 3D printing technology to achieve natural remodeling of the craniofacial region in a child.

**Conclusion:**

Large area of head and face missing structures can be reconstructed by using expanded skin flaps combined with 3D printing, and patients can get better new faces.

## Background

The head and face are important parts of the appearance and function of the human body. Because of the obvious location, children lack self-protection consciousness and are vulnerable to injury, which can cause many obstacles in the social life of such patients. Children are in the growth stage, and scars have to stabilize for a long time; once scar contracture occurs, it will restrict development. Reconstruction of the craniofacial region can be quite difficult for plastic surgeons. The advent of skin and soft tissue dilators is a milestone in the plastic surgery field. The expanded flap plays a superior role in aesthetic remodeling and functional recovery. Local scalp dilatation can easily correct scar baldness deformity. Due to the lack of adjacent tissue, large deformities affecting the whole face cannot be reconstructed simply by the expanded local flap; thus, an expanded axial-pattern flap is required. A supraclavicular perforator flap pedicled with the cervical cutaneous branch of the transverse cervical artery is a neurovascular pedicled flap with an ideal donor site for whole-face reconstruction. It is important to choose the right technique and apply a comprehensive approach to treat head and face deformities in developing children. 3D printing technology can provide a more complete and personalized clinical treatment plan. We could use 3D printing technology to repair skull defect and achieve skull remodeling. In this study, through the perfect combination of the expanded flap and 3D printing technology, a large-area missing structures of a child’s head and face was successfully repaired.

## Case presentation

**Fig. 1:**
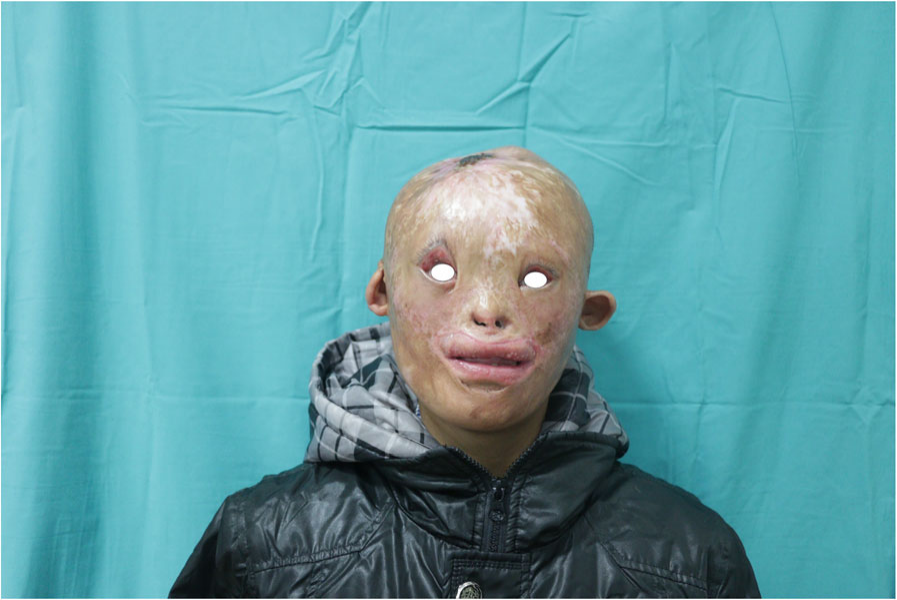
Photograph on admission showing scars after severe burns on the head and face, repeated ulceration at the top of the skull, and corneal leukoplakia

**Fig. 2:**
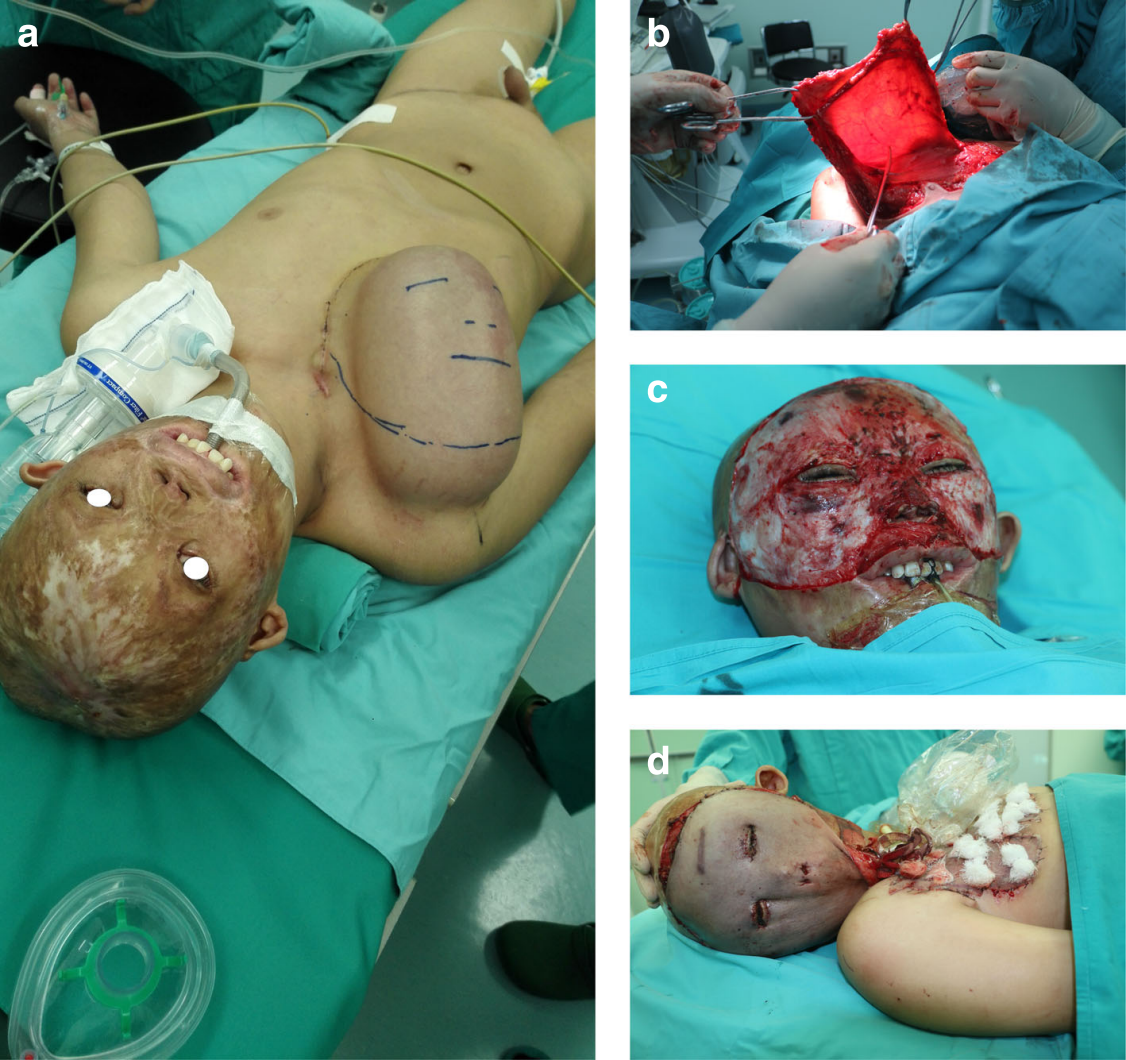
**a** Cutting lines drawn at the positions corresponding to the facial incisions above on the flap. **b** Light transmission experiment revealing the cervical transverse artery neck segment. **c** Removal of facial scars above the bilateral corners of the mouth. **d** Transfer of expanded axial flap with pedicle

**Fig. 3:**
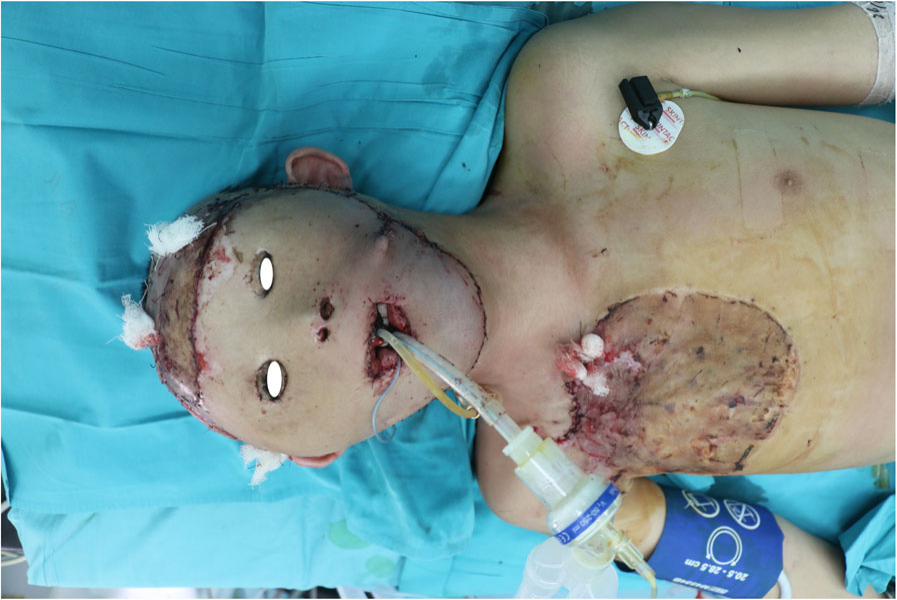
Repair of the lower facial area with the pedicled flap

**Fig. 4:**
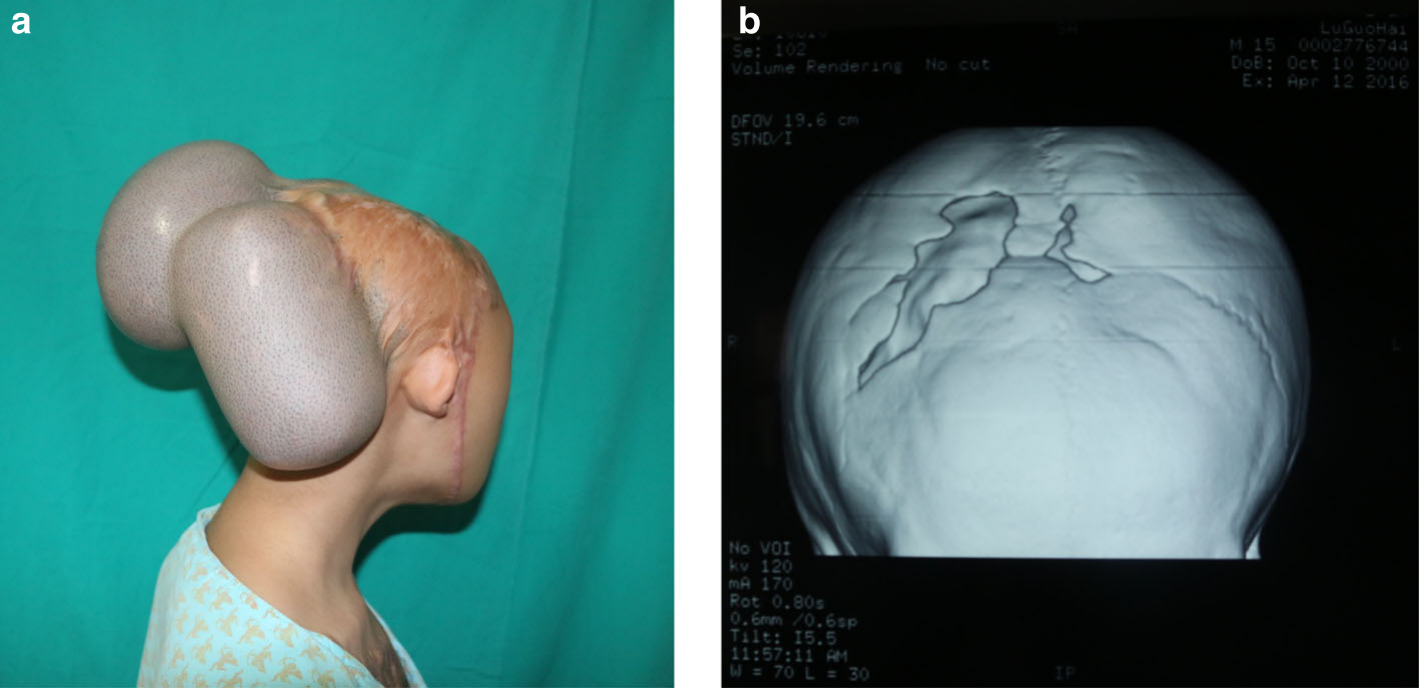
**a** Two embedded occipital dilators. **b** CT reconstruction showing the circumscribed bony defects

**Fig. 5:**
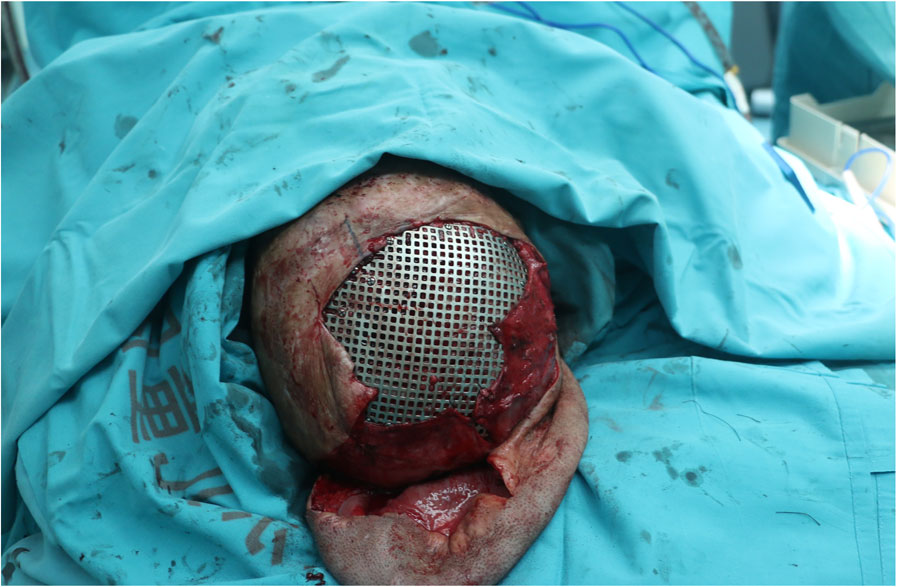
Placement of the preoperatively 3D-printed titanium mesh to repair the skull defect

A 15-year-old child was admitted to our department with severe scars in the craniofacial region in December 2015 (Fig. 1). When he was 4 months old, the child’s head and face were severely burned due to an accidental fire at home, resulting in extensive disfiguring scars. Due to family financial difficulties, the child did not receive timely treatment. Over the years, the scars on the top of the head have repeatedly broken. He was afraid to go out because of his ugly appearance. Volunteers found him, encouraged him to contact a foundation for financial support, and sent him to our department for head and face reconstruction. On admission, his eyelids failed to close due to severe cicatricial valgus, resulting in corneal leukoplakia and visual impairment. He underwent eight plastic surgery procedures in 10 months. The first operation was performed to implant an 800-ml dilator in his right chest and to release the scars on the eyelids and apply an autologous skin graft, which temporarily solved the problem of him not being able to close his eyes. After an inflating period of 4 months, the flap was delayed in the second operation. One week later, the third operation was performed. A Doppler flowmeter was used to determine the course of the cervical cutaneous branch of the transverse cervical artery, which was then marked on the surface of the skin. After removing the expander, the perforator skin flap was turned upward to repair the facial scar above the bilateral oral horn and perform autologous skin grafting around the pedicle (Fig. 2a, b, c, d). Perforations and sutures were applied where the eyes and nostrils were to be located on the skin flaps. At approximately 2 weeks after the third operation, the pedicle of the supraclavicular perforator flap was cut off and transferred to resurface the face below the mouth corners (Fig. 3). After half a month, the fifth operation was performed to further open the eyelids and corners of the mouth. A sixth operation was performed after 1 week. A prosthesis was implanted in the nose, and a skin graft was placed on the forehead wound. After the facial plastic surgery was completed, in May 2016, we placed two dilators on the top of the head and the occipital part of the patient (Fig. 4a and b). After 4 and a half months of inflation, the eighth operation was performed in collaboration with a neurosurgeon (Fig. 5). According to the preoperative CT reconstruction of the skull, a titanium mesh for repairing the skull defect was printed. The dilator was removed during the operation, and the neurosurgeon used a titanium nail to fix the titanium mesh. We covered the wound with a well-expanded scalp flap.

**Fig. 6:**
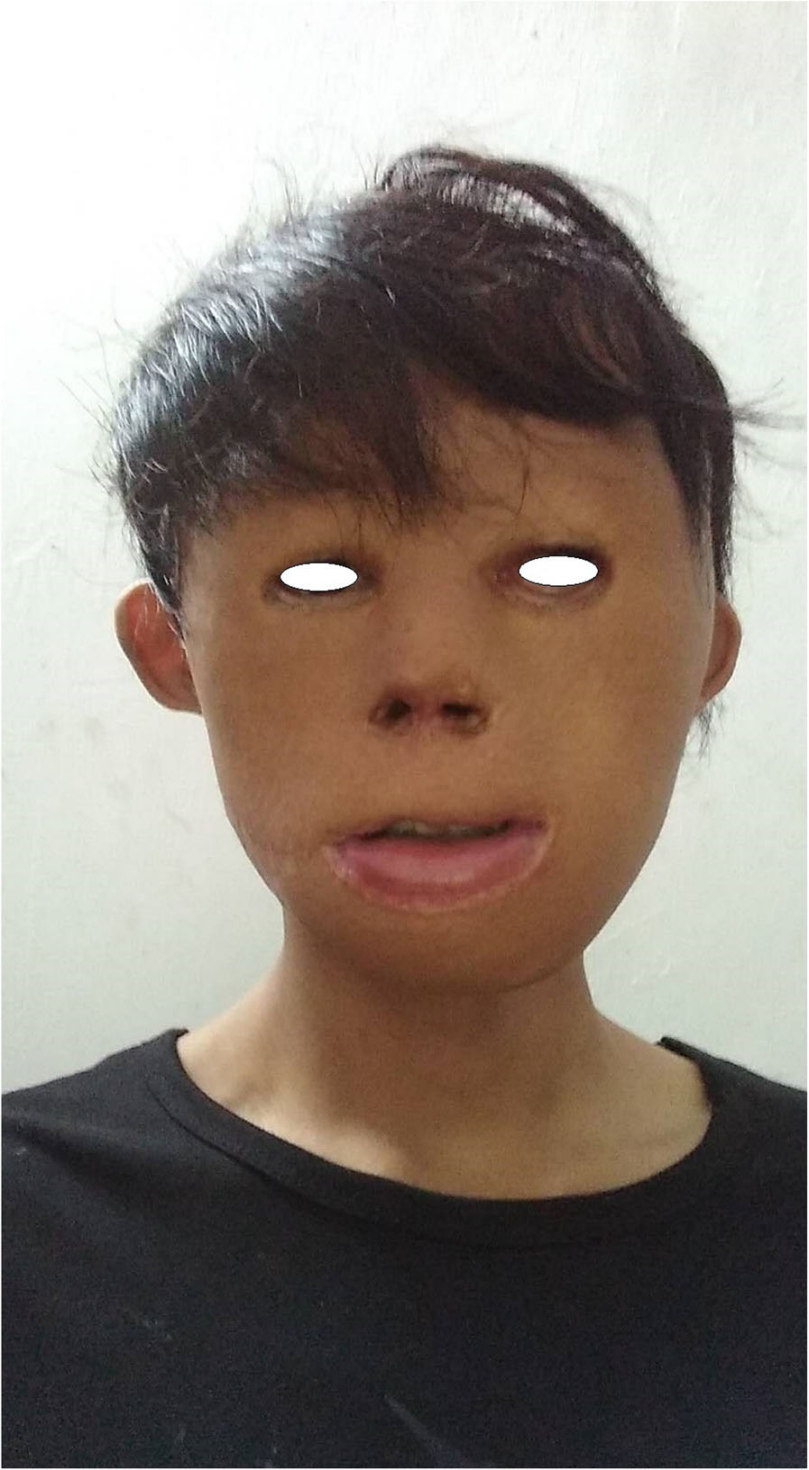
Follow-up photograph 3 years after the operations

Skin necrosis occurred at the margin of the reconstructed prefrontal region at the distal end of the flap, and all the other flaps and skin grafts survived. After 3 years of follow-up, the child’s head and face have achieved satisfactory aesthetic remodeling and functional recovery (Fig. 6).

## Discussion and conclusions

It is very common for plastic surgeons to encounter craniofacial structures missing, which can be quite challenging and should be repaired with good results. Skin grafts, pedicled transfer skin flaps, and free flaps have been applied in the treatment of craniofacial structures missing. For growing children, skin grafts are prone to severe scarring after transplantation, and may require multiple subsequent surgical procedures. Free skin flap transplantation has a relatively high failure rate due to the small blood vessels and difficult operation. Soft tissue expansion is the most ideal method for repairing large head and face structures missing. This approach can provide abundant skin with a similar color and texture and largely without conspicuous donor site morbidity.

Pre-expanded axial flaps have been clinically studied for a long time, and perforating flaps are a special type of axial flap. According to anatomical studies, human skin and subcutaneous tissue have an average of 374 large perforating vessels, as confirmed by three-dimensional and four-dimensional CT angiography and ultrasound Doppler angiography [1]. This means that all parts of the body can be used as a donor area for perforator flaps to repair large-area skin and soft tissue defects and not only restore the function of the injured area but also achieve the goal of aesthetic repair. In 1988, Kroll and Rosenfield pioneered the clinical application of perforating flaps [2]. In 2003, Tsai combined tissue expansion, microsurgery and perforator flaps to prefabricate free anterolateral thigh flaps for repairing neck scar contracture, which led to the study of the combination of the tissue expansion technique and perforator flaps [3]. According to the MLT principle proposed by Li et al. [4], it is better to consider matching the color and texture (M), having a large size graft (L) and harvesting thin skin tissue (T) in the selection of donor sites. The subcutaneous fat layer of clavicular and anterior thoracic skin is thin, and the skin color and texture are similar to those of the face; thus, these are suitable donor sites for facial repair. In this case, all of the facial features needed to be reconstructed. Therefore, it was appropriate to choose adjacent donor sites for harvesting superthin skin flaps. The surgical design involved perforator flaps from the supraclavicular region. The cutaneous branches of the transverse carotid artery were selected as pedicles to transfer the adjacent pedicled axial skin flaps. Pre-expanded perforator flaps are thinner and have good tissue compliance and could thus be used to realize partial reconstruction of the facial tissue. For the first time, the upper face above the bilateral corners of the mouth was repaired and the lower face below the bilateral corners of the mouth were repaired at the same time after the pedicle was cut off. The donor site was closed by skin grafting because the tissue around the larger donor site could not be pulled together and sutured. The eyes, nostrils and mouth were formed using incisions and sutures in stages. After the operation, the patient was transferred to the PICU ward. After he awakened from anesthesia, tracheal intubation was removed, and an oropharyngeal airway was placed to assist breathing. The blood supply of the flaps was observed after the operation, and the pedicle was cut off after two to 3 weeks of survival. The poor blood supply at the distal edge of the flaps may be related to the wider distal part of the flaps. After expansion, the flaps obviously became thinner. In the later stage of expansion, it was observed that the capillary reaction time at the distal end of the flaps was delayed more than that at the pedicle. To ensure the safety of the expanded flaps, one to two delayed flaps were added to ensure flap survival. If combined with microsurgical techniques, the distal survival rate will be significantly improved.

This child has a partial loss of the skull and a collapse of the surrounding skull, with nearly half of his hair lost, and the scalp ulcer did not heal for a long time. After facial reconstruction, the second major part of the operation was to reconstruct the appearance of the skull. Discussions between our department and the neurosurgery department determined that the scalp expanders should be implanted first, and then the defect of skull and scalp should be repaired in the second stage. The CT data were converted into 3D printing data before the operation, and a computer simulation was carried out. At the same time, the titanium mesh to be applied for repair was prepared by 3D printing technology before the operation. The skull was repaired by fixing the titanium mesh to the surrounding skull after peeling back the scalp. The computer-assisted personalized design of the titanium mesh not only repairs the patient’s skull loss and improves the recessed head shape. Thus, 3D printing technology effectively guided the operative process, allowed accurate completion of the scheduled operative plan, and effectively shortened the operative time. Similarly, this approach not only avoided the body temperature loss, brain tissue swelling and wound hemorrhage caused by prolonged dural exposure during the operation but also reduced the surgical trauma [5]. Pre-expanding the scalp adjacent to the skull defect area and covering the skull repair material with expanded scalp flaps effectively prevented the exposure-related complications of repair with common materials and repaired the scar baldness.

Some scholars have simplified the aesthetic divisions of the face into five regions, i.e., the forehead, cheek, perioral, nasal and periocular regions and carry out aesthetic restoration of facial subunits accordingly [6]. These operations completely corrected eyelid ectropion and cornual deviation, and made the eyes and mouth closed normally. The normal structure of the nose was completely replaced by scars, leaving only the nostrils for breathing. So we used prosthesis implantation to help restore the structure of the nose. These operations were performed to repair and replace the whole scar and improve the overall appearance. At the 3-year follow-up after the operation, it was apparent that the treatment had worked. The color and texture of the transferred skin flaps were similar to normal, and the scarring was light. Because of the deep tissue damage caused by burns in infancy, the expression after the repair is still unnatural. However, the patient was satisfied with the functional and visual recovery. The facial aesthetic subunits still lack elaborateness in design and require further plastic surgery.

In summary, skin and soft tissue expansion has become a routine clinical technique in plastic and aesthetic surgery because of its unique technical advantages and the various advantages of expanded skin flaps, which together provide a good method for repairing large-scale skin and soft tissue structures missing in children. Combining 3D printing technology, microsurgery technology, minimally invasive surgery technology and various new skin flap techniques will further promote the development of plastic and aesthetic surgery.

## Data Availability

All data generated or analysed during this study are included in this published article [and its supplementary information files].
